# Grade 3 Pulmonary Lymphomatoid Granulomatosis Diagnosed by Robotic‐Assisted Bronchoscopy: A Case Report

**DOI:** 10.1155/crpu/5840915

**Published:** 2026-03-09

**Authors:** Matthew Lee, Hussain Alkhafaji, Miguel Cantu, Kim Styrvoky, Steven Wu

**Affiliations:** ^1^ University of Texas Southwestern Medical School, University of Texas Southwestern Medical Center, Dallas, Texas, USA, utsouthwestern.edu; ^2^ Department of Pathology, University of Texas Southwestern Medical Center, Dallas, Texas, USA, utsouthwestern.edu; ^3^ Department of Surgical Oncology, Orlando Health Cancer Institute, Orlando, Florida, USA; ^4^ Department of Internal Medicine, University of Texas Southwestern Medical Center, Dallas, Texas, USA, utsouthwestern.edu

## Abstract

Lymphomatoid granulomatosis (LYG) is a rare, Epstein–Barr virus (EBV)–associated lymphoproliferative disorder characterized by angiocentric and angiodestructive infiltrates that most commonly involve the lungs. We report a case of a 38‐year‐old female with a history of latent tuberculosis who presented with a 3‐day history of fever, productive cough, bilateral lower extremity edema, and painless lower extremity skin lesions. Initial imaging revealed bilateral pulmonary nodules, and an extensive infectious workup was unrevealing. To obtain diagnostic tissue from peripheral lesions, she underwent robotic‐assisted bronchoscopy with targeted transbronchial biopsy. Histopathologic analysis revealed angiocentric and angiodestructive infiltrates with EBV positivity in CD20‐positive B cells, consistent with Grade 3 LYG. She was treated with dose‐adjusted etoposide, prednisone, vincristine, cyclophosphamide, doxorubicin, and rituximab (DA‐EPOCH‐R) chemotherapy, with initial radiographic response. However, she later developed refractory LYG complicated by hemophagocytic lymphohistiocytosis (HLH) and died of progressive disease. Robotic‐assisted bronchoscopy enabled safe, targeted sampling of peripheral pulmonary nodules, avoiding more invasive surgical biopsy.

## 1. Introduction

Lymphomatoid granulomatosis (LYG) is a rare, Epstein–Barr virus (EBV)–associated lymphoproliferative disease. This disease has a highly variable clinical presentation and may involve the lungs, skin, central nervous system (CNS), and other organs [1]. Patients commonly present with nonspecific symptoms such as cough, dyspnea, or constitutional symptoms [2]. LYG is a diagnostic challenge due to its rarity and overlapping features with opportunistic infections, autoimmune conditions, and other forms of malignancy.

The diagnosis of LYG is primarily driven by histopathologic analysis, which reveals atypical CD20‐positive B cells interspersed with CD3‐positive T lymphocytes in a mixed mononuclear infiltrate [3]. These infiltrates replace normal tissue architecture, causing vascular destruction and necrosis. The hallmark feature of LYG is its angiocentric and angiodestructive nature [4]. Other supporting diagnostic clues include Epstein–Barr virus–encoded RNA (EBER) positivity, multiple lung nodules on imaging, and skin or CNS involvement [3].

The World Health Organization (WHO) has classified LYG into three grades based on the number of EBV‐positive large B cells found on histological analysis. Grade 1 is defined as less than five EBV‐positive cells per high‐power field, whereas Grade 3 is defined as greater than 50 EBV‐positive cells per high‐power field [5]. We present a case of a 38‐year‐old female with systemic and pulmonary involvement of Grade 3 LYG. Because LYG is rare and can mimic common conditions, timely tissue diagnosis is essential; this case highlights robotic‐assisted bronchoscopy as an effective approach to obtain diagnostic pulmonary tissue in suspected LYG.

## 2. Case Presentation

A 38‐year‐old female healthcare worker with eczema and a history of latent tuberculosis (TB) treated with isoniazid presented with 3 days of fever, productive cough, bilateral lower extremity edema, and painless lower extremity skin lesions.

Chest radiography revealed bilateral pulmonary nodules. Contrast‐enhanced chest CT showed prominent pulmonary nodules scattered throughout both lungs with hilar lymphadenopathy (Figure 1). An infectious evaluation was performed, including QuantiFERON‐TB Gold testing, acid‐fast bacilli (AFB) stains, fungal serologies, and bacterial cultures. The differential diagnosis included mycobacterial infection, fungal infection, and malignancy, and pulmonology was consulted for tissue sampling.

Figure 1Chest CT before and after treatment demonstrating radiographic response: (a) Axial contrast‐enhanced chest CT showing a 2.3‐cm nodule in the anterolateral right lower lobe (arrow). (b) Axial noncontrast chest CT showing interval decrease of the same right lower lobe nodule to 1.1 cm (arrow). (c) Axial contrast‐enhanced chest CT showing a 3.0‐cm lobulated nodule in the left lower lobe (arrow). (d) Axial noncontrast chest CT showing interval decrease of the left lower lobe nodule to 1.5 cm (arrow).

**Figure:**
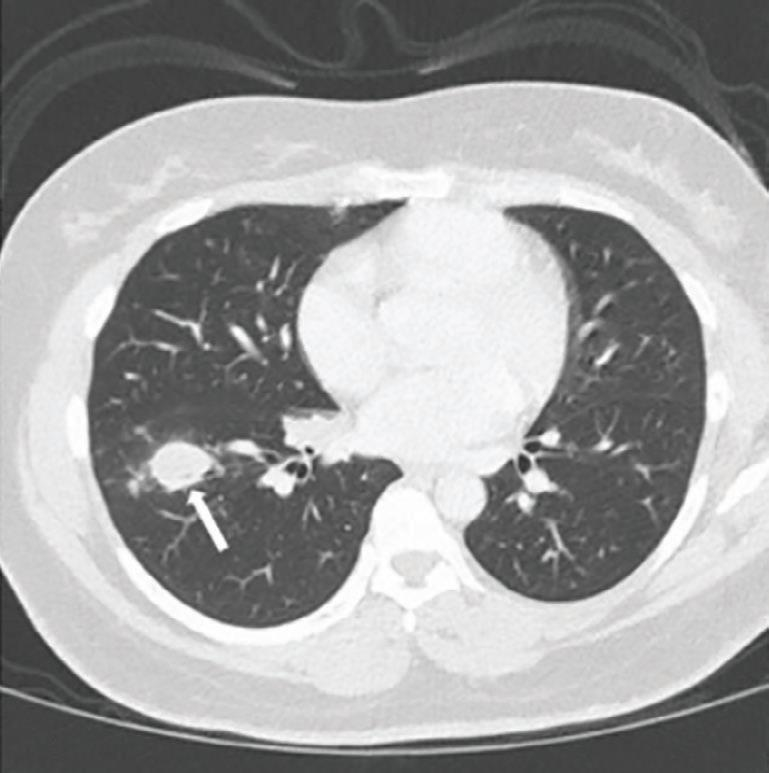
(a)

**Figure:**
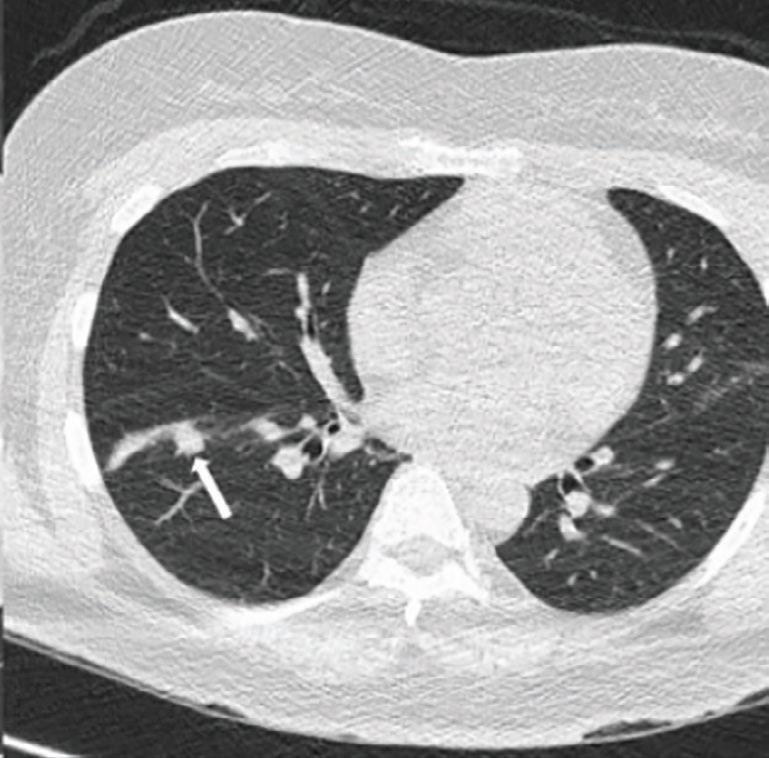
(b)

**Figure:**
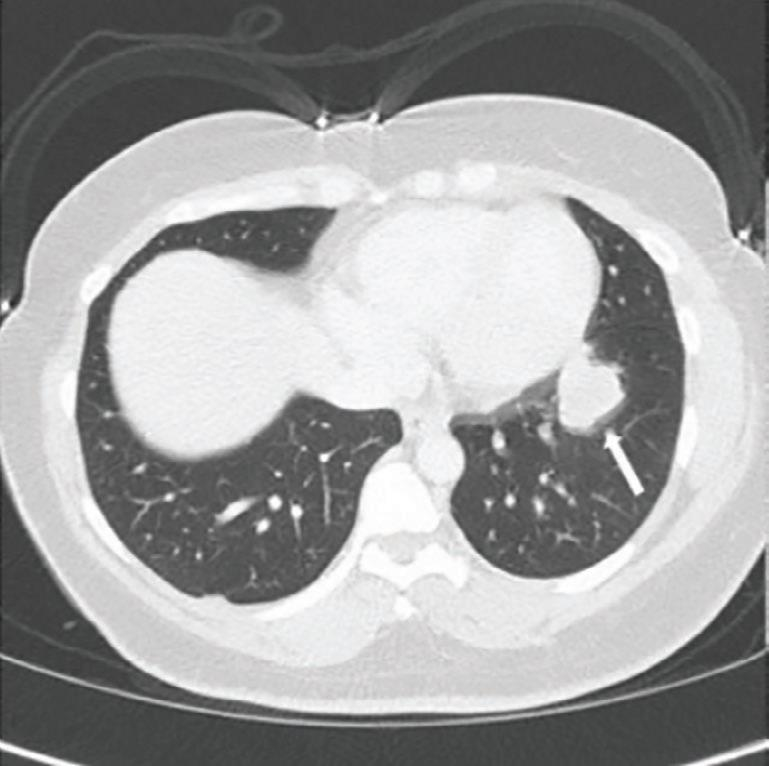
(c)

**Figure:**
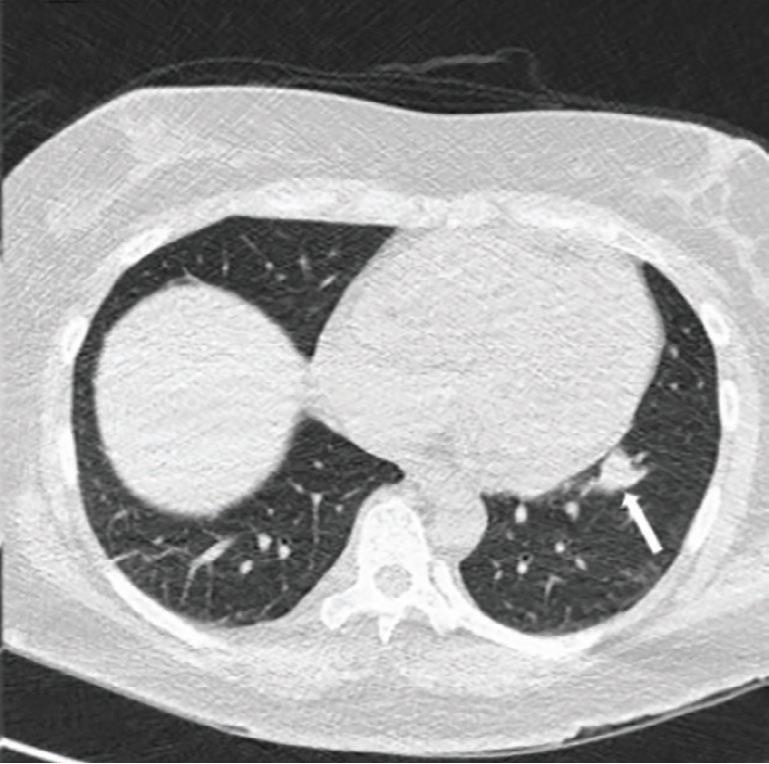
(d)

She underwent robotic‐assisted bronchoscopy using the ion endoluminal system with CT‐based planning. The right lower lobe (RLL) target was confirmed with fluoroscopy and radial endobronchial ultrasound (r‐EBUS). Transbronchial needle aspiration of the RLL mass was performed (four passes: one for cultures and three for cytology). Microforceps transbronchial biopsies were then obtained (12 passes: 1 for cultures and 11 for pathology). Bronchoalveolar lavage was also performed in the RLL. Lastly, an endobronchial lesion in the left upper lobe was biopsied with forceps (four samples). While awaiting final pathology and culture results, empiric rifampin, isoniazid, pyrazinamide, and ethambutol were initiated due to concern for possible TB reactivation. Moxifloxacin was added for potential drug‐resistant organisms. However, all infectious studies were ultimately unrevealing.

**Figure 2:**
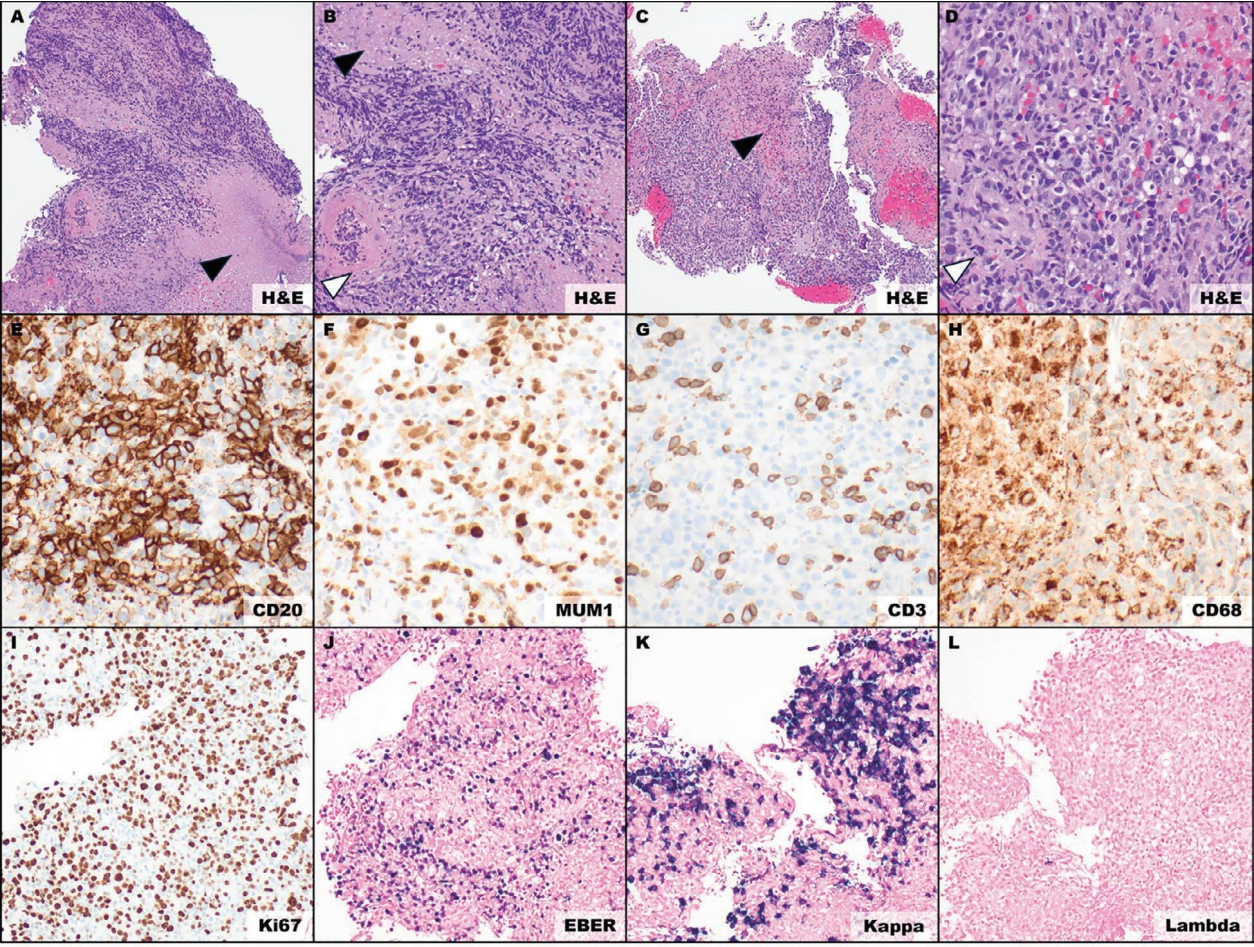
Histopathologic features of Grade 3 lymphomatoid granulomatosis. Biopsy specimens were obtained via robotic‐assisted bronchoscopy to safely target peripheral pulmonary nodules. (A, B) Left upper lobe endobronchial biopsy; (C–L) right lower lobe transbronchial biopsy; (A, B) mixed inflammatory cells with large atypical lymphoid cells, central necrosis, and angionecrosis ((A) 100x, (B) 200x); (C, D) mixed inflammatory cells with scattered large atypical lymphoid cells, necrosis, and angioinvasion ((C)100x, (D) 200x); (E) CD20‐positive large B cells, IHC (400x); (F) MUM1‐positive large B cells and plasma cells, IHC (400x); (G) CD3‐positive scattered small T cells, IHC (400x); (H) CD68‐positive scattered histiocytes, IHC (400x); (I) elevated Ki‐67 proliferation index, IHC (400x); (J) numerous EBV‐positive cells, EBER ISH (400x); (K) kappa immunoglobulin light chain restriction, ISH (400x); (L) lambda immunoglobulin light chain negative, ISH (400x). Black arrowhead—necrosis; white arrowhead—vascular necrosis and angioinvasion. Abbreviations: H&E, hematoxylin and eosin; CD, cluster of differentiation; IHC, immunohistochemistry; ISH, in situ hybridization; x, magnification.

Histopathologic examination from the RLL lung biopsy and the left upper lobe endobronchial biopsy revealed an EBV‐positive angiocentric lymphoid proliferation with transmural vessel involvement and vascular‐mediated necrosis. The infiltrate was polymorphous and included increased intermediate to large B cells. These B cells demonstrated kappa light‐chain restriction and were admixed with T cells, plasma cells, and histiocytes (Figure 2). EBER in situ hybridization showed 82 EBV‐positive large B cells per high‐power field, supporting Grade 3 LYG by WHO criteria [5].

**Figure 3:**
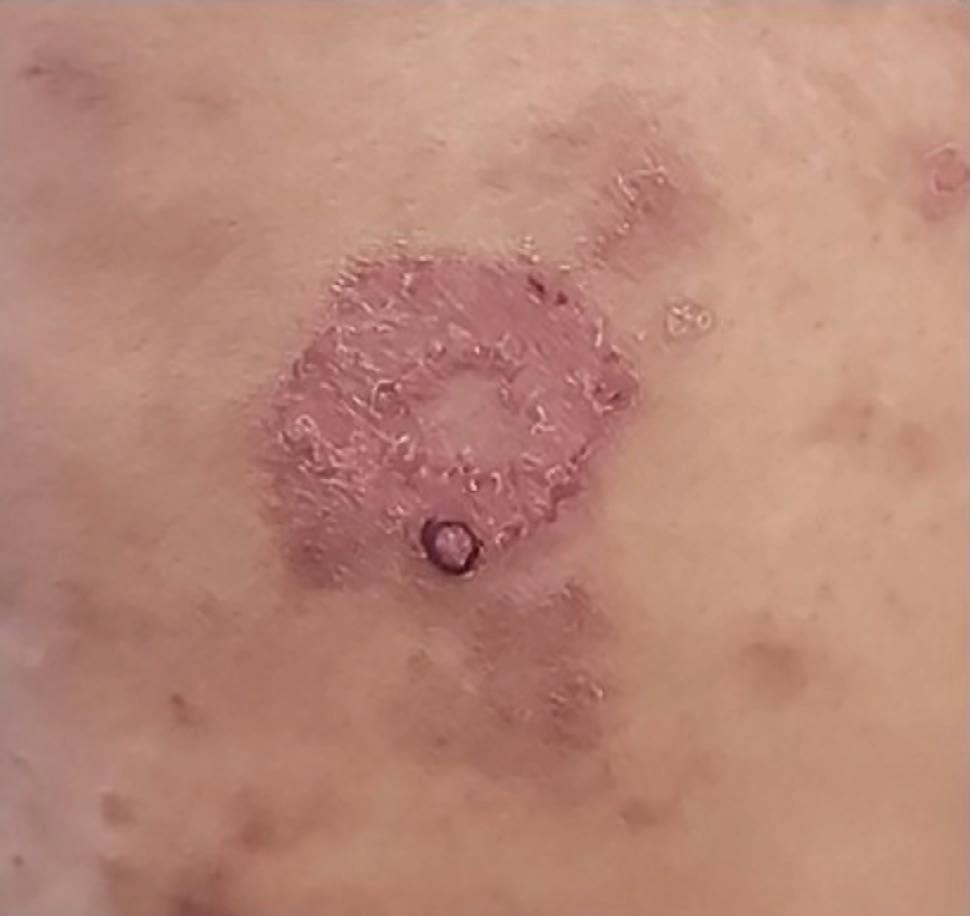
Clinical photograph of right thigh lesion: Right thigh lesion with a black circle marking the site of punch biopsy.

Her lower extremity skin lesions were initially presumed to be eczema (Figure 3) and treated with topical steroids. Given the lack of improvement, a punch biopsy of a right thigh lesion was performed. It revealed granulomatous inflammation without infectious organisms, consistent with the overall LYG diagnosis. For staging, PET/CT imaging revealed FDG‐avid pulmonary nodules, hilar lymphadenopathy, and multifocal FDG‐avid skin thickening in the lower extremities. A bone marrow biopsy showed no evidence of lymphoproliferative involvement.

The patient was admitted to oncology and initiated on dose‐adjusted etoposide, prednisone, vincristine, cyclophosphamide, doxorubicin, and rituximab (DA‐EPOCH‐R), with methotrexate for CNS prophylaxis. She completed six cycles of therapy, with intermittent febrile episodes. Follow‐up noncontrast chest CT demonstrated a radiographic response with decreased size of pulmonary nodules (Figure 1b,d).

Six months later, she developed hemophagocytic lymphohistiocytosis (HLH) with intrathoracic LYG progression. Her course was further complicated by *Pseudomonas* infection, and she ultimately died of progressive disease.

## 3. Discussion

Our case demonstrates the diagnostic challenges of LYG and the value of robotic‐assisted bronchoscopy in establishing the diagnosis. LYG is rare and most affects men (male‐to‐female ratio > 2:1) in their fourth to sixth decades of life [1, 6]. However, cases have also been documented in younger patients, including children [7]. LYG is an EBV‐driven lymphoproliferative disease that is thought to arise from impaired immune surveillance of EBV‐infected B cells [8–10]. A prospective study from the National Cancer Institute (NCI) found that all patients with LYG had at least moderately decreased T cell numbers, supporting immune dysfunction in its pathogenesis [8]. However, similar to our patient, many patients have no known immunodeficiency at presentation [9]. Therefore, the absence of known immunodeficiency should not exclude LYG from consideration [10].

LYG often presents with nonspecific respiratory and constitutional symptoms and can mimic more common infectious, inflammatory, and malignant conditions [1, 4]. In our patient, a history of treated latent TB and occupational healthcare exposure raised concern for mycobacterial reactivation. This prompted presumptive treatment and delayed definitive tissue diagnosis. The patient′s constellation of symptoms, including pulmonary nodules, skin lesions, and generalized malaise is also typical of TB infection, vasculitis, granulomatous infections, and sarcoidosis [11]. Malignancy, including lymphoma, also remained a key consideration. Despite a broad infectious workup, the diagnosis remained elusive until histopathologic analysis.

Pulmonary involvement is one of the key features of LYG. In a prospective NCI cohort, 100% of patients had pulmonary involvement, and the lung was the most frequently biopsied site (73%) [1]. Accordingly, pulmonary tissue sampling is central to diagnosis. In our patient, histopathologic analysis of pulmonary tissue revealed angiocentric and angiodestructive infiltrates with EBV‐positive, atypical CD20‐positive B cells admixed with CD3‐positive T cells, plasma cells, and histiocytes, confirming the diagnosis of LYG (Figure 2).

Cutaneous involvement is also common and is reported in approximately one‐third of patients at presentation [1, 12]. Skin lesions are often nonspecific and can mimic systemic diseases such as sarcoidosis and granulomatosis with polyangiitis [13, 14]. In our patient, numerous painless lower extremity lesions were initially attributed to eczema and treated with topical corticosteroids (Figure 3). A skin biopsy showed granulomatous inflammation with a T cell predominant infiltrate and was EBV‐negative. Immunohistochemical staining showed most lymphocytes were CD3‐positive, with only a few CD20‐positive B cells, and EBER was negative. Although granulomatous inflammation may be seen in cutaneous LYG, skin biopsies often lack the pathognomonic features of CD20‐positive B cells and EBV detection. Thus, skin biopsy may be nondiagnostic despite active systemic disease [15].

In contrast, pulmonary tissue most reliably demonstrates the hallmark histopathologic findings of LYG [1]. Obtaining diagnostic pulmonary tissue can be difficult when nodules are peripheral or multifocal. Robotic‐assisted bronchoscopy is advantageous in this setting because it improves navigation to distal airways and provides catheter articulation and stability during biopsy [16]. It also has a strong safety profile and good diagnostic performance in peripheral lesions [16, 17]. Diagnostic yield can be improved with tool‐in‐lesion confirmation, including r‐EBUS and cone‐beam CT (CBCT) [16, 17]. Compared with conventional guided bronchoscopy (e.g., electromagnetic navigation bronchoscopy), robotic‐assisted bronchoscopy has demonstrated higher diagnostic yields in meta‐analyses (up to ~84%) [17]. In our patient, robotic‐assisted bronchoscopy enabled safe, targeted sampling of peripheral nodules and provided optimal tissue for definitive histopathologic analysis.

Because LYG is a lymphoproliferative disorder, its clinical and radiographic features can resemble high‐grade lymphoma, including EBV‐positive diffuse large B cell lymphoma (DLBCL) [8]. EBV‐positive DLBCL typically shows diffuse sheets of monomorphic large B cells. It is generally considered a diagnosis of exclusion when criteria for other EBV‐positive B cell lymphoproliferative disorders are not met [5, 18]. In our case, the presence of extranodal lung and skin lesions, angiocentricity with transmural vessel involvement, polymorphous inflammatory background, and 82 EBV‐positive B cells per high‐power field supported a diagnosis of Grade 3 LYG by WHO criteria [5]. Bone marrow biopsy may be performed to assess for hematologic involvement and to exclude alternative lymphoid malignancies [1, 8]. In our patient, a bone marrow biopsy showed no evidence of lymphoproliferative involvement. This finding aligns with existing literature demonstrating that bone marrow disease is rare in LYG [1, 19].

There is currently no consensus on the treatment of LYG. In low‐grade disease, interferon alfa‐2b or observation may be warranted, as low‐grade LYG is more immune‐dependent and can occasionally enter spontaneous remission [4, 19]. Patients with Grade 3 disease, such as our patient, require immediate therapy due to its aggressive nature. It also carries the potential to progress to malignant lymphoma, reported in approximately 12% of cases [20]. Combination chemotherapy with regimens like DA‐EPOCH‐R has shown efficacy in Grade 3 LYG, with a prospective trial reporting that 47% of patients achieving complete remission after initial therapy. However, the 5‐year progression‐free survival was only 25.4%, highlighting the high risk of relapse associated with this disease [19].

Our patient initially demonstrated radiographic response to DA‐EPOCH‐R but later developed refractory LYG complicated by secondary HLH. HLH is a life‐threatening hyperinflammatory syndrome, most often triggered by infections such as EBV or malignancy [21]. EBV‐driven disease can precipitate secondary HLH by sustaining cytotoxic T cell and macrophage activation, leading to excess cytokine production [22, 23]. However, HLH associated with LYG appears to be rare, with only a few cases reported [24]. Our patient′s clinical course underscores the need for vigilant long‐term monitoring of high‐grade LYG given its risk of relapse, progression, and life‐threatening complications such as HLH.

## 4. Conclusion

Our case of Grade 3 LYG demonstrates the diagnostic and management challenges associated with this rare condition. Robotic‐assisted bronchoscopy with targeted biopsies enables timely histopathologic examination, which is essential for establishing the diagnosis. A coordinated multidisciplinary approach is vital for diagnosis and treatment. Given the substantial risk of relapse and severe complications such as HLH, vigilant long‐term surveillance is essential for disease management.

## Funding

No funding was received for this manuscript.

## Ethics Statement

Ethics approval was not required for this case report in accordance with the policies of the University of Texas Southwestern Medical Center.

## Consent

Verbal informed consent for publication of this case and accompanying images was obtained from the patient prior to her passing. The case has been sufficiently anonymized in accordance with ICMJE guidelines. All efforts have been made to ensure this report does not contain any identifying information.

## Conflicts of Interest

The authors declare no conflicts of interest.

## Data Availability

Data sharing is not applicable to this article as no datasets were generated or analyzed during the current study.
